# Mechanism of Action of *Streptococcus downii*, a New Bacterial Species with Probiotic Potential

**DOI:** 10.3390/antibiotics12091472

**Published:** 2023-09-21

**Authors:** Lucía Martínez-Lamas, Eliane García-Mato, Anniris Rincón-Quintero, Berta Rivas-Mundiña, Pedro Diz-Dios, Maximiliano Álvarez-Fernández

**Affiliations:** 1 Clinical Microbiology, Hospital Álvaro Cunqueiro, Complejo Hospitalario Universitario de Vigo, Microbiology and Infectology Group, Galicia Sur Health Research Institute (IISGS), 36212 Vigo, Spain; lucia.martinez.lamas@sergas.es (L.M.-L.); anniris.maria.rincon.quintero@sergas.es (A.R.-Q.); maximiliano.alvarez.fernandez@sergas.es (M.Á.-F.); 2 Medical-Surgical Dentistry Research Group (OMEQUI), Health Research Institute of Santiago de Compostela (IDIS), University of Santiago de Compostela (USC), 15782 Santiago de Compostela, Spain; eliane.garma@gmail.com (E.G.-M.); berta.rivas@usc.es (B.R.-M.)

**Keywords:** bacteriocin, probiotic, dental caries, *Streptococcus mutans*, *Streptococcus downii*

## Abstract

*Streptococcus downii* is a recently reported bacterial species of oral origin, with inhibitory capacity against *Streptococcus mutans*, *Actinomyces naeslundii*, *Veillonella parvula* and *Aggregatibacter actinomycetemcomitans*, which confers upon it the potential of being an oral probiotic. The aim of the present study was to identify the potential mechanisms by which *S. downii* exerts its inhibitory effect on *S. mutans*. To this end, the study assessed the consumption of glucose and proteins available in the culture medium, the modification of the pH, the production of short-chain fatty acids, the changes in the protein panel of the inhibition halo, the production of hydrogen peroxide and the effect of proteinase K. There were no differences in the glucose values or in the protein content of the medium, but there was a reduction in pH (with no effect on the growth of *S. mutans*). Significant increases were detected in the levels of lactic and formic acid (with no effect on the growth of *S. mutans*), as well as changes in the peptide panel (with no effect on the growth of *S. mutans*). The inhibitory effect was maintained in the presence of peroxidase but disappeared after adding proteinase K. Based on these results, it is suggested that the main mechanism of inhibition of *S. downii* against *S. mutans* is the production of bacteriocins.

## 1. Introduction

Analysis of oral microbiota using next-generation sequencing techniques has enabled the identification of differing clusters of microbial communities that are dependent on oral health, thereby suggesting that good oral health corresponds to a combination of innocuous commensal species that make up a stable ecosystem and hinder the growth of pathogenic bacteria [1,2,3]. Any alteration to this homeostasis, which maintains a healthy microbial composition, results in a microbial dysbiosis that can lead to oral diseases such as dental caries and periodontal disease [2]. The strategies for modulating the dental biofilm include promoting the growth of beneficial bacteria by administering pre- and probiotics [4].

Probiotics are living microorganisms that, when administered in the appropriate quantities, are able to produce a health benefit in the host [5]. The use of probiotics is based on bacterial competition processes [6,7,8] that can occur either directly, due to the secretion of bacteriocins, toxins, enzymes or waste products, or indirectly via changes in the environmental pH [6,9]. These microorganisms stimulate cell-mediated immunity, occupy the niches of pathogenic microorganisms and interrupt host–pathogen communication, thereby progressively replacing pathogenic strains [10]. Although the exact mechanism of probiotic activity in the oral cavity is not fully understood, it is considered to be the result of a combination of the local and system immune responses together with other, non-immunological mechanisms [11,12,13].

*Streptococcus downii* is a new bacterial species initially detected as part of the oral microbiome in a teenager with Down syndrome with no history of dental caries. *S. downii* has shown inhibitory capacity against *Streptococcus mutans*, *Actinomyces naeslundii*, *Veillonella parvula* and *Aggregatibacter actinomycetemcomitans*. In a biofilm model, *S. downii* showed an antibiofilm effect against *S. mutans* and a significant reduction in *S. mutans* and *Aggregatibacter actinomycetemcomitans* counts and significantly reduced the growth of *P. gingivalis* and *V. parvula* in well-structured biofilms [14,15].

Given that *S. mutans* is the main bacterial species involved in the onset and progress of dental caries [16], the aim of this study was to identify the possible mechanism of action by which *S. downii* exerts its inhibitory effect on *S. mutans*.

## 2. Results

### 2.1. Availability of Carbon Source and Protein Content

A comparison of solubilized agar samples obtained from a plate of *S. mutans* ATCC (American Type Culture Collection) 25175^T^ prior to culturing *S. downii* and those obtained from the inhibition halo generated by *S. downii* after culture with *S. mutans* ATCC 25175^T^ showed that the mean protein contents were 6.363 ± 0.110 and 6.640 ± 0.100 mg/mL (*p* = 0.1), respectively, with a mean glucose content of 6.377 ± 0.070 and 7.620 ± 0.080 g/L (*p* = 0.1), respectively.

### 2.2. Modification of Environmental Potential of Hydrogen (pH)

The pH levels detected in distinct growth environments varied between the medium for the *S. mutans* ATCC 25175^T^ plate with no *S. downii* and the medium for the inhibition halo generated by *S. downii* after culture in the presence of *S. mutans* ATCC 25175^T^, with mean pH values of 6.667 ± 0.321 and 5.500 ± 0.200 (*p* = 0.1), respectively.

To determine whether this acidification was responsible for the growth inhibition of *S. mutans* ATCC 25175^T^, the growth ability of this bacterial strain was studied at a pH of 5.5, finding that the decrease in pH was not responsible for the inhibition.

### 2.3. Organic Acid Production

Table 1 shows the differential production of organic acids in the solubilized agar samples obtained from the *S. mutans* ATCC 25175^T^ plate before culture with *S. downii* and those obtained from the inhibition halo generated by *S. downii* after culture with *S. mutans* ATCC 25175^T^.

Higher levels of lactic (27-fold) and formic acid (10-fold) were detected in the samples obtained from the inhibition halo of *S. downii* than in those obtained prior to culture with *S. downii*, although these results did not reach statistical significance. In light of these findings, the direct inhibitory effect of both acids on *S. mutans* ATCC 25175^T^ was investigated by diluting the acids to various concentrations (formic acid: 0.01, 0.03, 0.06, 0.12 and 0.24 mg/mL; lactic acid: 0.05, 0.10, 0.20, 0.40, 0.80 and 1.6 mg/mL). A combination of 0.24 mg/mL of formic acid and 1.6 mg/mL of lactic acid was also tested. After adding 5 µL of each acid to *S. mutans* ATCC 25175^T^ and incubating for 17 h, no inhibition was observed at any concentration or after adding the combination of both organic acids. Consequently, the production of short-chain organic acids by *S. downii* was ruled out as being responsible for the inhibition of *S. mutans* ATCC 25175^T^.

### 2.4. Analysis of Protein Fractionation

Solubilized agar samples collected before culture with *S. downii* and from the inhibition halo generated after culture thereof with *S. mutans* ATCC 25175^T^ were analyzed, fractionating the protein content by applying cut-offs with a molecular size of 3 and 10 kDa. The fractionated protein content < 3 kDa was 3.190 ± 0.165 and 2.107 ± 0.200 (*p* = 0.1), respectively, for both sample types, while the content < 10 kDa was 5.017 ± 0.101 and 3.733 ± 0.208 (*p* = 0.1), respectively.

Although the growth of *S. downii* caused changes in the peptide profile, the inhibitory effect was not reproduced when inoculating these fractions directly onto *S. mutans* ATCC 25175^T^. Consequently, the presence of peptide compounds in the <3 kDa and <10 kDa fractions produced by *S. downii* with a potential ability to inhibit *S. mutans* ATCC 25175^T^ was ruled out.

### 2.5. Hydrogen Peroxide Production and Proteinase K Test

**Figure 1:**
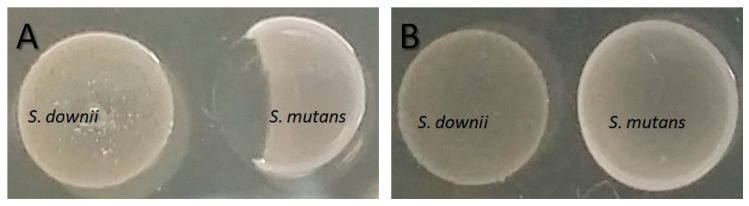
Inhibition test for *S. downii* versus *S. mutans* in the presence of peroxidase (**A**) and proteinase K (**B**). Hydrogen peroxide production is not responsible for the inhibition, given that after adding peroxidase, the inhibitory capacity of *S. downii* versus *S. mutans* is maintained (**A**); however, the presence of proteinase K eliminates the inhibitory effect, which confirms the molecule’s protein nature with inhibitory capacity produced by *S. downii* (**B**).

Figure 1 shows the result of the presence of peroxidase and proteinase K on the inhibitory effect produced by *S. downii* on *S. mutans* ATCC 25175^T^. This inhibitory effect is maintained in the presence of peroxidase but disappears after adding proteinase K, suggesting that inhibition might be caused by a molecule of a peptide-type nature.

### 2.6. Inhibition of S. mutans by the S. downii Supernatant

**Figure 2:**
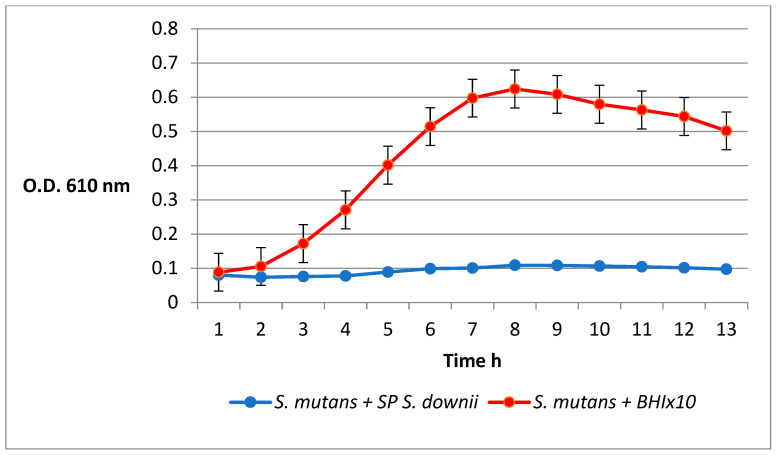
Growth curves of *S. mutans* in the presence of concentrated *S. downii* supernatant (SP *S. downii*) and concentrated BHI medium (BHI × 10). The addition of the concentrated *S. downii* supernatant inhibits the growth of *S. mutans* (blue line), while *S. mutans* preserves its growth capacity after the addition of the concentrated BHI medium (red line). Mean and standard deviation of three replicates.

Figure 2 shows how the addition of the concentrated *S. downii* supernatant produces an inhibitory effect against the growth of *S. mutans* compared with the growth curve obtained when adding the concentrated brain heart infusion (BHI) medium.

**Figure 3:**
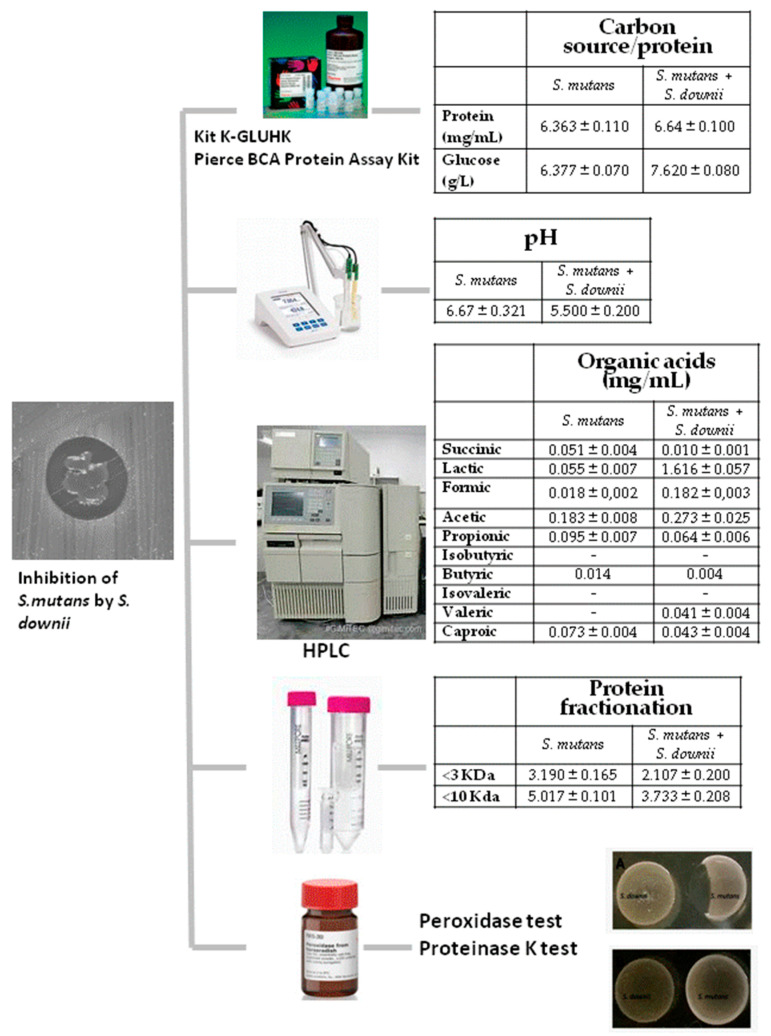
Summary of the experiments performed to study the mechanism of *S. downii* inhibition of *S. mutans*.

In summary, there were no differences in the glucose values or in the protein content of the medium, but there was a reduction in pH (with no effect on the growth of *S. mutans*). Significant increases were detected in the levels of lactic and formic acid (with no effect on the growth of *S. mutans*), as well as changes in the peptide panel (with no effect on the growth of *S. mutans*). The inhibitory effect was maintained in the presence of peroxidase but disappeared after adding proteinase K, suggesting the presence of the inhibitory molecules in the *S. downii* supernatant. Figure 3 summarizes all the experiments performed to study the mechanism of *S. downii* inhibition of *S. mutans* as well as the results.

## 3. Discussion

The known mechanisms by which probiotics inhibit and/or interfere with other bacterial populations include pH modification, the production of antimicrobial compounds, competition for binding sites and/or nutrients and the stimulation of immunomodulatory cells [17].

Given that the binding of microorganisms to the tooth surface represents the first step in the pathogenesis of dental caries, competition during this adhesion process as a result of bacterial adhesion is one of the key mechanisms of action for oral probiotics [18]. Indeed, in vitro studies have shown the ability of various species of the genus *Streptococcus* to inhibit the colonization of epithelial cells by *A. actinomycetemcomitans* [19]. Similarly, *S. mitis* inhibits the adhesion of *Porphyromonas gingivalis* to gingival epithelial cells [20], and *S. dentisani* inhibits the growth of periodontal pathogens by way of adherence, competition and displacement mechanisms [21].

Bacterial competition for the available substrates is another possible inhibition mechanism. *Bifidobacterium* spp. has been observed to interrupt the growth of *P. gingivalis* by reducing the essential nutritional factors available in the environment [15].

The production of alkaline substances might prevent growth of the pathogens responsible for dental caries and alter the chemical equilibrium in favor of tooth remineralization. Somewhat paradoxically, the production of organic acids, such as lactic and acetic acid, causes a decrease in pH as the non-dissociated form of the acid enters the bacterial cell, where it dissociates in the cytoplasm, thereby acidifying the medium. This intracellular build-up of the ionized form of the acid could itself cause the death of the potential pathogen [22,23].

Another mechanism exhibited by oral commensal streptococci and that affects the ecosystem of the oral biofilm due to its inhibitory effect on certain microorganisms is the production of hydrogen peroxide (H_2_O_2_) from lactic acid, thereby minimizing the decrease in pH. H_2_O_2_ inhibits the growth of *S. mutans* and numerous other oral pathogens at concentrations that do not markedly affect the producing strains [24]. Lactic acid bacteria produce various antimicrobial agents, including organic acids, H_2_O_2_, low molecular weight antimicrobial peptides, bacteriocins and adhesion inhibitors [25,26]. Polonskaia [27] was the first author to claim that *Lactobacillus acidophilus* might inhibit the growth of other bacteria. Specifically, *Lactobacilli* from the strain GG produce organic acids, H_2_O_2_, carbon dioxide, diacetyl, low molecular weight antimicrobial peptides, bacteriocins and adhesion inhibitors against *Streptococcus* spp. and can therefore be considered a potent probiotic [28]. *S. sanguinis*, which is a H_2_O_2_ producer, has been found to suppress the growth of *A. actinomycetemcomitans* in vitro and to antagonize the colonization thereof in gnotobiotic rats [29]. Similarly, Tong et al. and Bao et al. showed that *S. oligofermentans* inhibits the growth of *S. mutans* by producing H_2_O_2_, both in suspension and in biofilm studies [30,31]. *Streptococcus* A12 also inhibits growth and intercellular signaling in *S. mutans* and can exert a buffer effect by modifying the pH via the arginolytic pathway [32].

However, the percentage contribution of lactic acid and other organic acids to the antibacterial activity of lactic acid bacteria has been estimated at less than 50% [33]. Terai et al. showed that *Lactobacillus* spp. isolates conserved some antibacterial activity after being neutralized by these acids, suggesting that the isolates produce bacteriocins or other antibacterial substances [34]. Indeed, bacteriocins have been isolated from certain *Lactobacillus* spp., such as salivaricin from *L. salivarius* [35], reuterin and reutericyclin from *L. reuteri* [36] or plantaricin from a strain of *L. plantarum* [37]. Reuterin is a broad-spectrum antimicrobial agent that can inhibit the growth of Gram-positive and Gram-negative bacteria, including oral pathogens such as *S. mutans*, *A. actinomycetemcomitans*, *Prevotella intermedia* and *Fusobacterium nucleatum* [38]. A strain of *S. mutans* (JH1001) that produces a bacteriocin, namely mutacin 1140, which inhibits the growth of a wide range of bacteria, including *Streptococcus* spp., *Actinomyces* spp. and *Lactobacillus* spp. in vitro, has been isolated [39,40,41]. Previously, the genome for the JH strain of *S. salivarius* has been shown to contain the biosynthetic loci for the bacteriocins salivaricin A3 and E, streptin, and streptococcin SA-FF22; this strain also produces the bacteriocin zoocin A, which, in combination with the preparation of dextranase, exhibited potent anti-*S. mutans* activity [42]. The strain *S. salivarius* K12, in turn, produces salivaricin, which is mainly responsible for its potential as an oral probiotic [43]. Similarly, the probiotic *S. salivarius* M18 produced bacteriocins that antagonize the acidogenic activity of *S. mutans*. When this strain colonizes the human oral mucosa, it produces dextranase and urease, which can counteract plaque formation and the acidity of saliva, respectively [44]. *S. dentisani*, in turn, metabolizes arginine and produces bacteriocins to exert its oral probiotic effect [45,46].

In light of the above, we can consider at least three mechanisms by which *S. downii* exerts its probiotic action: competition for the binding site to oral tissues and/or nutrients, the production of antimicrobial agents and/or bacteriocins and modification of the host’s immune response. Given that the experiments in this study were performed in vitro, we were unable to demonstrate a role for immunomodulation in the inhibitory effect observed.

No major differences in nutrient content were detected for the solubilized agar samples obtained from the *S. mutans* ATCC 25175^T^ plate prior to culture with *S. downii* and those obtained from the inhibition halo generated by *S. downii* after culture with *S. mutans* ATCC 25175^T^. It therefore appears unlikely that *S. mutans* inhibition is the result of competition for the substrate or a depletion of nutrients. Although differences in terms of pH were observed after culture with *S. downii*, the effect thereof on the growth of *S. mutans* was ruled out in subsequent studies. A marked increase in the levels of lactic acid and formic acid was detected in the samples obtained from the inhibition halo generated by *S. downii* after culture with *S. mutans* ATCC 25175^T^ compared with the solubilized agar samples obtained from the *S. mutans* ATCC 25175^T^ plate prior to culture with *S. downii*. However, neither of these two acids exhibited a direct inhibitory effect on *S. mutans*. H_2_O_2_ production was also ruled out as a mechanism for bacterial antagonism, given that the inhibitory effect remained unchanged after adding peroxidase.

Given that inhibition in the in vitro tests occurred after pre-growth of the strain, *S. downii* either produces a metabolite that diffuses through the agar or modifies the medium, thereby favoring *S. mutans* inhibition. Consequently, the most likely option to explain the inhibitory effect observed is the production of antimicrobial substances of a peptide-type nature, which can diffuse freely through the agar, thereby inhibiting the growth of susceptible strains [14]. The likelihood that the inhibitory molecules are peptidic in nature is strengthened by the fact that the inhibitory effect was significantly reduced upon treatment with proteinase K [47].

Based on the results and given that a significant number of *Streptococcus* spp. isolates were shown to be bacteriocin-producing [48], class IIb bacteriocins have been identified in the genomes of *Streptococcus pyogenes* [49], *Streptococcus* MIA (M 18) [50] and *S. dentisani* [51], among others. In this sense, it has been pointed out that the genomic analysis of *S. downii* has revealed the presence of putative genes of the family of bacteriocin IIb lactobinA/cerein 7B [52]. A comparative genomics study of *S. downii* to detect the presence of these genes related to the production of bacteriocins would confirm the study’s findings. Moreover, it is essential to isolate the bacteriocin through the concentrated supernatant, similar to the study by Conrads et al. with *S. dentisani* [51].

## 4. Materials and Methods

### 4.1. Inhibition of S. mutans by S. downii

A 0.3 McFarland suspension of *S. mutans* ATCC 25175^T^ was prepared on BHI plates to obtain a carpet-type growth. A 10-µL aliquot of *S. downii* was deposited on each plate in the form of a button after completing growth in pure culture for 24 h. *S. mitis* ATCC 49456T and *S. salivarius* ATCC 7073T were used as negative controls, and *S. dentisani* CECT 7747^T^ was used as a positive control. The plates were incubated at 35–37 °C in an aerobic atmosphere for 24 h. The inhibitory capacity versus *S. mutans* ATCC 25175^T^ was confirmed at 48 h by the presence of inhibition haloes. The test was performed in triplicate.

### 4.2. Inhibition of S. mutans by the S. downii Supernatant

To obtain the concentrated supernatant, we inoculated several colonies of *S. downii* in 50 mL of brain heart infusion broth (BHI, Bd) incubating at 37 °C for 48 h in an aerobic atmosphere. After incubation, the mixture was centrifuged at 4000 rpm for 10 min, discarding the pellets. The supernatant was passed through filters (pore size of 0.2 μm) (Millipore) and concentrated in a Rotavapor (rotary evaporator) 10 times. The resulting 5 mL was once again passed through filters (pore size of 0.2 μm) (Millipore) and stored at −20 °C until its use.

The supernatant’s inhibitory activity was determined by measuring the absorption at 610 nm in an automated microplate reader, incubating the sample at 37 °C for 14 h and recording the absorption every 30 min. To this end, we diluted *S. mutans* with BHI medium until an optical density of 0.1 was achieved. We then mixed 160 μL of the *S. mutans* suspension with 40 μL of the concentrated *S. downii* supernatant. As a negative control, we mixed 160 μL of the *S. mutans* suspension with 40 μL of concentrated BHI 10 times. The experiment was repeated 3 times.

### 4.3. Availability of Carbon Source and Protein Content

To confirm that the inhibition of *S. mutans* ATCC 25175^T^ did not occur as a result of the consumption of glucose or proteins present in the medium by *S. downii*, agar samples (some lacking *S. downii* and others from the inhibition halo region that appears in the plate tests after culturing *S. downii* for 17 h) were collected, solubilized and analyzed. Glucose was analyzed using the biochemical kit K-GLUHK (Megazyme, Bray, Ireland). Proteins were analyzed using a Pierce BCA Protein Assay Kit (Thermo Scientific, Rockford, IL, USA).

The agar solubilization protocol was performed as described previously with minor modifications [53]. Using a set of parallel blades, we obtained 3 strips (measuring 5mm × 5 mm) of BHI agar from the *S. mutans* plaque before culture with *S. downii* and from the inhibition halo generated by the co-culture of both species. Each fragment of agar medium was added to 1.5 mL of double-distilled sterile water and melted in a bath at 85–90 °C for 3 min (the agar’s melting temperature). We then measured the resulting volume to be able to adjust the calculations according to the dilution factor obtained in each case. We used as copies equidistant fragments of the *S. mutans* plaque before culture with *S. downii* and those obtained from the inhibition halo generated by the co-culture of both species.

### 4.4. Modification of Environmental pH

To study whether pH modification of the medium caused by the growth of *S. downii* affected the growth of *S. mutans* ATCC 25175^T^, agar samples were taken, solubilized in a similar manner to those used in the nutrient-consumption experiments at various growth points and in the inhibition zone generated, and analyzed to detect any differences in terms of pH.

### 4.5. Organic Acid Production

Certain short-chain organic acids, such as succinic, lactic, formic, acetic, propionic, isobutyric, butyric, isovaleric, valeric and caproic, might inhibit the growth of *S. mutans* ATCC 25175^T^. To analyze the differential production of these organic acids, two samples of solubilized agar (one with no *S. downii* and the other from the inhibition halo generated by this microorganism after culture with *S. mutans* ATCC 25175^T^) were taken. The organic acids present therein were analyzed by high-performance liquid chromatography coupled to a refractive index detector [54]. The direct action of these acids on *S. mutans* ATCC 25175^T^ at supposedly inhibitory concentrations was also studied. To that end, the acids were diluted in phosphate-buffered saline (PBS) and a 5-µL aliquot inoculated to *S. mutans* ATCC 25175^T^ simultaneously.

### 4.6. Analysis of Protein Fractionation

To evaluate the possible changes in the protein (peptide) profile after the culture of *S. downii*, the protein content of two solubilized agar samples was analyzed. One sample lacked *S. downii*, and the other was obtained from the inhibition halo generated after the culture of *S. downii* together with *S. mutans* ATCC 25175^T^. Samples were ultrafiltered on an exclusion column (Amicon, Millipore, Bedford, MA, USA) of 3 and 10 kDa (molecular weight cut-offs), and the protein content was quantified using the Pierce BCA Protein Assay Kit (Thermo Scientific, Rockford, IL, USA). Both protein fractions were also tested directly, adding 5 µL of each while simultaneously inoculating *S. mutans* ATCC 25175^T^.

### 4.7. Hydrogen Peroxide Production and Proteinase K Test

To evaluate the production of H_2_O_2_ by *S. downii*, after incubation in a solid medium for 24 h, 10 μL of peroxidase (64 μg/mL) was added to one side of the colony, and 10 μL of PBS was added to the other. Subsequently, 5 μL of *S. mutans* ATCC 25175^T^ was inoculated to the right and left sides, as reported previously by other authors [6,55]. *S. sanguinis* DMSZ (German Collection of Microorganims and Cell Cultures) 20567^T^ was used as a positive control [56], and *S. mutans* ATCC 25175^T^ was used as a negative control. If *S. downii* is a H₂O₂-producer, slight effervescence is observed immediately, and no inhibition haloes are observed after 24 h.

To determine whether bacteriocins might be responsible for the inhibition by *S. downii*, after incubation in a solid medium for 24 h, 10 μL of proteinase K (70 μg/mL; Sigma, St. Louis, MO, USA) was added to one side of the colony, and 10 μL of PBS was added to the other. Subsequently, 5 μL of *S. mutans* ATCC 25175^T^ was inoculated to the right and left sides, as reported previously by other authors [6,18]. Mutacin-producing *S. mutans* ATCC 25175^T^ [6] was used as a positive control, and *S. sanguinis* DMSZ 20567^T^ was used as a negative control. The plates were visualized after incubation at 37 °C for 24 h, and the absence of haloes indicated that inhibition was caused by molecules of a peptide-type nature.

### 4.8. Statistical Analysis

For the statistical analysis of the experimental data, we employed the statistical package SPSS (SPSS 28.0 for Windows, SPSS Ltd., Surrey, United Kingdom). Due to the non-normal distribution, the differences between the mean values were assessed using the nonparametric Mann–Whitney U test. Significance was established for *p* ≤ 0.05. All experiments have been repeated 3 times, and we agree that the statistical power of 3 samples is very weak, and therefore these results should be interpreted with extreme caution.

## 5. Conclusions

The results of this study show that the mechanism of action by which *S. downii* exerts its inhibitory effect on *S. mutans* is not regulated by the consumption of glucose and proteins available in the culture medium, the change in pH, the production of short-chain fatty acids, the changes in the inhibition halo’s protein panel or the production of hydrogen peroxide. Consequently, and given that the inhibitory effect disappeared after adding proteinase K, these results allow us to speculate that the most likely mechanism by which *S. downii* exerts its inhibitory action on certain oral pathogenic bacteria is the production of antimicrobial substances of a peptide nature.

## Figures and Tables

**Table 1:** Mean and standard deviation concentration of three replicates of organic acids present in solubilized agar samples obtained from the *S. mutans* ATCC 25175^T^ plate before culture with *S. downii* (*S. mutans*) and those obtained from the inhibition halo generated by *S. downii* after culture with *S. mutans* ATCC 25175^T^.

	Organic Acids (mg/mL)		
	*S. mutans*	*S. mutans* + *S. downii*	*p*-Value
Succinic	0.051 ± 0.004	0.010 ± 0.001	0.1
Lactic	0.055 ± 0.007	1.616 ± 0.057	0.1
Formic	0.018 ± 0.002	0.182 ± 0.003	0.1
Acetic	0.183 ± 0.008	0.273 ± 0.025	0.1
Propionic	0.095 ± 0.007	0.064 ± 0.006	0.1
Isobutyric	-	-	-
Butyric	0.014	0.004	-
Isovaleric	-	-	-
Valeric	-	0.041 ± 0.004	-
Caproic	0.073 ± 0.004	0.043 ± 0.004	0.1

## Data Availability

The data are available upon request to the corresponding author.
